# The *in vitro* Effects of the Probiotic Strain, *Lactobacillus casei* ZX633 on Gut Microbiota Composition in Infants With Diarrhea

**DOI:** 10.3389/fcimb.2020.576185

**Published:** 2020-09-10

**Authors:** Xing Wang, Miao Zhang, Weidong Wang, Haoxin Lv, Hua Zhang, Yuan Liu, Zhongfang Tan

**Affiliations:** ^1^Henan Key Laboratory of Ion-Beam Bioengineering, School of Agricultural Sciences, Zhengzhou University, Zhengzhou, China; ^2^The Third Affiliated Hospital Xinxiang Medical University, Xinxiang, China; ^3^School of Food Science and Technology, Henan University of Technology, Zhengzhou, China; ^4^School of Food and Biological Engineering, Henan University of Animal Husbandry and Economy, Zhengzhou, China

**Keywords:** *Lactobacillus casei*, infant, diarrhea, gut microbiota, high-throughput sequencing

## Abstract

We investigated the *in vitro* effects of *Lactobacillus casei* ZX633 on gut microorganism composition in infants with diarrhea. For this purpose, 103 feces samples from healthy infants (healthy group) and 300 diarrhea samples from infants (diarrhea group) were collected, and diarrhea feces were treated with *L. casei* ZX633, which was previously isolated from healthy infant feces (treatment group). We used microbial dilution plate methods, high performance liquid chromatography (HPLC) and high-throughput sequencing approaches to analyze viable main microorganism counts, short chain fatty acid (SCFA) concentrations, and intestinal microbiota composition in feces, respectively. Our data showed that *L. casei* ZX633 supplementation increased the numbers of *Escherichia coli*, yeasts, lactic acid bacteria (LAB) and aerobic-bacteria, raised propionic acid levels but reduced four other SCFAs, which are close to the healthy group. Alpha diversity results indicated that microbial diversity and richness decreased in treatment group. Bacterial community analyses revealed that microbial structures of the treatment group tended toward the healthy group; i.e., *Escherichia-Shigella* and *Clostridioides* abundance increased, and there was a reduction in the abundance of *Streptococcus, Bacteroides, Enterococcus* and *Veillonella*. In conclusion, *L. casei* ZX633 isolated from healthy infant feces, may be effective in improving infant diarrhea microbiota, potentially providing a new probiotic strain to reduce the incidence of diarrhea associated with bacterial disease in infants.

## Introduction

Diarrhea is defined as the passage of three or more loose liquid stools per day, whereas a diarrheal duration of 14 days constitutes acute or persistent diarrhea (Walker et al., 2013). This condition is an enormous health problem for children under 5 years old, and globally, is related to significant morbidity and mortality rates (Wen et al., 2018). Annually, there are approximately 1.7 billon cases of childhood diarrhea disease, with 760,000 deaths, thus representing the second leading cause of death in children (Gallardo et al., 2017).

Diarrhea arises from infectious diarrhea due to bacterial, viral and parasitic organisms, and non-infectious diarrhea, derived from malnutrition and food allergies (Shin and Shin, 2018). *Escherichia coli, Salmonella enteritidis, Shigella*, and *Staphylococcus aureus* are the main common pathogens that cause bacterial diarrhea (Wang et al., 2018). Generally, the bacterial condition is treated with antibiotics, however, antibiotic use is considered a double-edged sword, as adverse effects can become evident with antibiotic usage, especially antibiotic resistance. Previous study has shown that antibiotic misuse is a severe problem, causing compositional and richness changes in intestinal microbiota, potentially detrimental to host health (Pilla et al., 2019).

Increasingly, interest has turned toward natural alternatives, as a result of antibiotic resistant effects and restrictions on the uncontrolled use of antibiotics. One alternative treatment for bacterial diarrhea are probiotics, natural and live microorganisms which when administered in adequate amounts confer a health benefit on the host (Hill et al., 2014). They improve the intestinal microbiota balance by competitive inhibition of the colonization of pathogenic bacteria in the intestinal tract (Bubnov et al., 2015; Bautista-Gallego et al., 2019; Wongsen et al., 2019). In addition, they also have beneficial effects on the gut immune system (Bubnov et al., 2015). Current evidence suggests that some probiotics such as *Lactobacillus* and *Bifidobacteria* modulate gut microflora homeostasis, and may exert protective effects against diarrhea (Kwok et al., 2014, 2015). *Lactobacillus rhamnosus* GG notably reduced diarrheal duration in children upon receiving a dosage no <10^10^ colony-forming units (CFU) per day (Li et al., 2019), whereas *Lactobacillus casei* increased *Bifidobacteria* and *Lactobacillus* counts in stool and improved diarrhea in infants (Lai et al., 2019). In addition, lactic acid bacteria (LAB) from fecal origins have been used as potential probiotics for diarrhea, and are considered promising since these bacteria have long time associations with humans (Gheziel et al., 2019). Therefore, a LAB strain was isolated from healthy infant feces and used as a reagent in this study.

The main study aim was to investigate whether *L. casei* ZX633 supplementation improved diarrhea in infants. Using *in vitro* approaches, we assessed differences in fecal microflora in healthy, diarrhea and diarrhea feces supplemented with *L. casei* ZX633. Our data may provide a new probiotic strain for treating diarrhea associated with bacterial disease.

## Materials and Methods

### Sample Collection

All experimental procedures in this study were approved by the Institutional Ethics Committee of Zhengzhou University. Prior to the collection of the children's stool and data, consent was given by their parents. A total of 403 fecal samples (300 diarrhea fecal samples and 103 healthy fecal samples) were collected from infants <5 years old at the Third Affiliated Hospital of Xinxiang Medical University, China. Before the study, we selected diarrhea fecal samples with high leukocyte counts which reflect a bacterial infection. Samples were kept at 4°C, and transported under the same conditions to Zhengzhou University, where they were stored at −80°C until required.

### Bacterial Strains and Treatment of Diarrhea Stool Samples

*L. casei* ZX633 was isolated from the feces of a healthy infant with strong antimicrobial activities, which had reported in our previous research (Wang et al., 2020) (Table 1). The strain was cultured on Man Rogosa Sharpe agar (MRS, Merck, Darmstadt, Germany) at 30°C, under anaerobic conditions for 48 h. Diarrhea samples (1 g) were homogenized in 200 μl *L. casei* ZX633 fermentation broth (previously cultured in MRS broth at 30°C for ~8 h), using a vortex mixer to reach a LAB viable cell concentration of approximately 10^5^ CFU/g. The mixture was then incubated at 37°C for 48 h (Treatment).

**Table 1:** *L. casei* ZX633 characteristics.

Basic properties	10^°^C	w
	30^°^C	++
	45^°^C	–
	pH3.5	w
	pH4	+
	pH9	+
	3% NaCl	++
	6.5% NaCl	+
Antibiotic susceptibility	CT, CN, P, VA, CIP	Resistant
	TE, E, C, RD, AMP	Susceptible
Antibacterial activity	*E. coli* ATCC 30105	++
	*S. enterica* ATCC 13076	+++
	*Ps. aeruginosa* ATCC 27853	+++
	*M. luteus* ATCC 4698	++++
	*S. aureus* ATCC 29213	++

*Basic properties: ++, grew very well; +, grew well; w, grew weakly; – no growth*.

*Antibiotic susceptibility: Antibiotic concentrations are expressed in μg/disc; Ampicillin (AMP, 10), Gentamicin (CN, 10), Erythromycin (E, 15), Vancomycin (VA, 30), Chloramphenicol (C, 30), Tetracycline (TE, 30), Penicillin (P, 10), Rifampicin (RD, 5), Ciprofloxacin (CIP, 5), Colistin sulfate (CT, 10)*.

*Antibacterial activity: The inhibition zone was measured to the external diameter of the cup; –, no inhibition; +, 10–15 mm; ++, 15–20 mm; +++, 20–25 mm; ++++, >25 mm*.

### Isolation and Identification of LAB Strains

Samples were homogenized in sterile water using a vortex mixer. After this, 10-fold, 10^3^-fold, and 10^5^-fold dilutions were made and plated onto MRS agar, then grown for 48 h at 30°C under anaerobic conditions. One representative colony of each morphology type (different size, shape and/or color) was picked and streaked onto fresh MRS agar. Pure cultures, with catalase negative activity, were identified by genetic analysis using PCR and 16S rRNA sequencing. The universal PCR primers, 27F (5′-AGAGTTTGATCCTGGCTCAG-3′) and 1492R (5′-GGTTACCTTGTTACGACTT-3′) (Zhang et al., 2016) were used to amplify the 16S rRNA gene. PCR parameters consisted of an initial heating at 95°C for 3 min; followed by 35 cycles of 15 s at 95°C, 15 s at 60°C and 90 s at 72°C, and a final extension at 72°C for 5 min. PCR products were sequenced by the Huada Biotech Company (Zhengzhou, China), and compared to strains in the GenBank database using BLAST, on the NCBI website (https://blast.ncbi.nlm.nih.gov/Blast.cgi).

### Microbial Counting

Microbial communities were counted by the dilution plate procedure, according to Zhang et al. (2017). Samples (0.1 g) were suspended in 900 μl sterile water and mixed. Ten-fold and serial dilutions (10^−2^ to 10^−5^) were then prepared in sterile water. Then, 20 μl of each dilution dilutions was spread onto various selection agar plates.

Bacterial colony numbers were counted, based on different selection media and colony appearance. LAB colonies were counted on MRS agar after incubation at 30°C for 48 h, under anaerobic conditions. Aerobic bacteria, yeasts and *E. coli* were cultured on nutrient agar (NA), potato dextrose agar (PDA) with 10% dihydroxysuccinic acid solution (final concentration 1.5%), and eosin-methylene blue medium (EMB) at 37°C for 48 h, respectively. Separately, 10^−1^ and 10^−2^ dilutions were spread onto NA and *Clostridium difficile* agar (CLO) to ascertain *Bacillus subtilis* (37°C for 48 h) and *C. difficile* (37°C for 48 h under anaerobic conditions) growth, after dilutions were incubated for 15 min at 75°C. Colonies were counted, and the logarithmic numbers of viable colony-forming units in fecal samples (log CFU/g) were calculated. Three replicates were analyzed.

### Quantification of Short Chain Fatty Acid (SCFA) in Fecal Samples

SCFAs including lactic, formic, acetic, propanoic and butyric acid were measured by high performance liquid chromatography (HPLC), using a Waters Alliance series 2695 (Zhang et al., 2018). Fecal samples were centrifuged (6,000 rpm for 10 min) and supernatants were filtered through a 0.22 μm pore-size filter (Jin Teng, Zhengzhou, China) prior to analysis. HPLC conditions were: a Carbomix H-NP10 column (7.8 × 300 mm); mobile phase, 2.5 mmol/l H_2_SO_4_; flow rate, 0.6 ml/min; temperature: 55°C. SCFA levels were determined from the area under the peak, detected at 240 nm using a calibration curve.

### DNA Extraction and PCR Amplification

Total DNA was extracted from fecal samples using the bacterial DNA Kit (D3350-02, Omega Biotek, Norcross, GA, USA). After extraction, DNA integrity and quality was checked by 1% agarose gel electrophoresis and a NanoDrop 2000 UV-Vis spectrophotometer (Thermo Scientific, Wilmington, USA). PCR reaction were performed in 20 μl volumes, containing 4 μl Fast pfu buffer, 2 μl 2.5 mM dNTPs, 0.8 μl each primer (5 μM), 0.4 μl Fast pfu polymerase, 0.2 μl bovine serum albumin (BSA) and 10 ng template DNA. The V3–V4 regions of bacterial 16S rDNA was amplified with using the primers, 338F (5′-ACTCCTACGGGAGGCAGCAG-3′) and 806R (5′-GGACTACHVGGGTWTCTAAT-3′) (Yang et al., 2019). PCR parameters were 3 min denaturation at 95°C, 29 cycles of 30 s at 95°C, 30 s annealing at 53°C, 45 s elongation at 72°C and a final extension at 72°C for 10 min.

### Illumina MiSeq Sequencing

PCR products were sequenced on an Illumina MiSeq PE300 platform (Shanghai Majorbio Bio-pharm Technology Co. Ltd., China), which measured diversity and bacterial composition in infant feces.

### Statistical Analysis

LAB and microbial communities, and SCFA levels were analyzed using IBM SPSS Statistics 25.0 and Origin 2017. A one-way analysis of variance (ANOVA) was used to compare the means with a significant difference at *p* < 0.05. Data from high throughput sequencing were analyzed on the free online platform, Majorbio Cloud Platform (http://www.majorbio.com). Sequences were de-multiplexed and quality-filtered using the QIIME (version 1.9.1 http://qiime.org/install/index.html), and the obtained high quality reads were clustered into Operational taxonomic units (OTUs) using UPARSE (version 7.0 http://www.drive5.com/uparse/), at 97% similarity. Chao, Ace, Shannon, Simpson index and Good's coverage were computed using Mothur (version 1.30.2 https://www.mothur.org/wiki/Download_mothur) for analysis of alpha-diversities. The similarity and differences between samples were compared using the shared and unique OTUs of the Venn diagram. The taxonomy of each 16S rRNA gene sequence was analyzed by Ribosome Database Project (RDP) Classifier (version 2.11 http://rdp.cme.msu.edu/) against the Silva (SSU 132) 16S rRNA database. Spearman correlations were used to analyze the SCFAs and bacterial relationships using the pheatmap package.

## Results

### LAB Strain Differences Between Diarrhea and Healthy Fecal Samples

**Figure 1:**
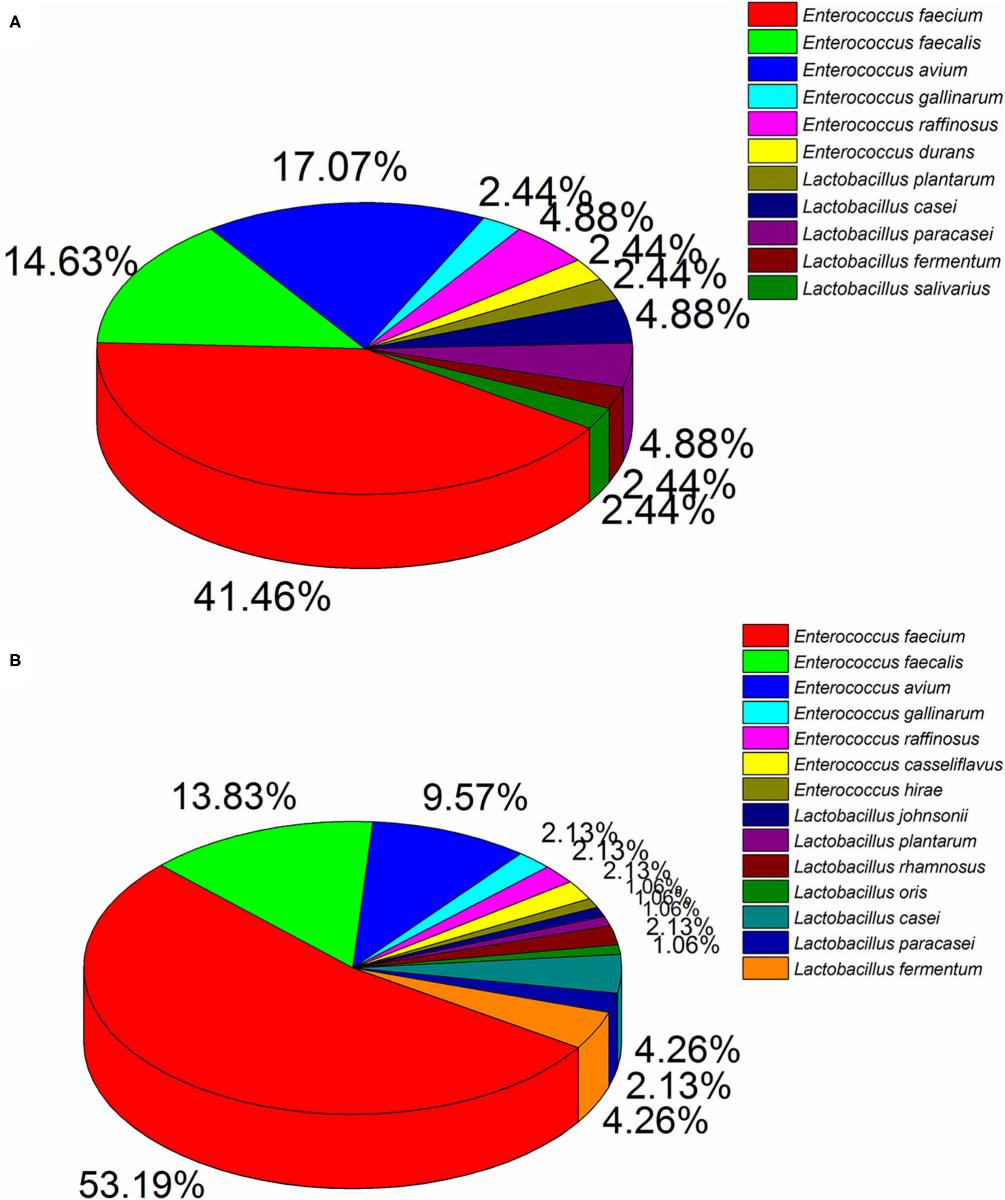
Dominant LAB strains in healthy **(A)** and diarrhea **(B)** feces.

Ninety four and 41 LAB strains were isolated from diarrhea and healthy infant fecal samples, respectively. According to 16S rDNA sequencing analyses (Figure 1), *Enterococcus* was the predomination LAB. Healthy feces comprised mainly *E. faecium* (41.46%), *E. avium* (17.07%), *E. faecalis* (14.63%), and *E. raffinosus* (4.88%), whereas diarrhea feces comprised mostly *E. faecium* (53.19%), *E. faecalis* (13.83%), and *E. avium* (9.57%). Similarly, members of the *Lactobacillus* genera were also detected, of which *L. casei* (4.88%), *L. paracasei* (4.88%), *L. fermentum* (2.44%), and *L. plantarum* (2.44%) were primarily found in healthy feces, and *L. casei* (4.26%), *L. fermentum* (4.26%), *L. paracasei* (2.13%), and *L. rhamnosus* (2.13%) were most common in diarrhea feces.

### Microbial Counts

**Figure 2:**
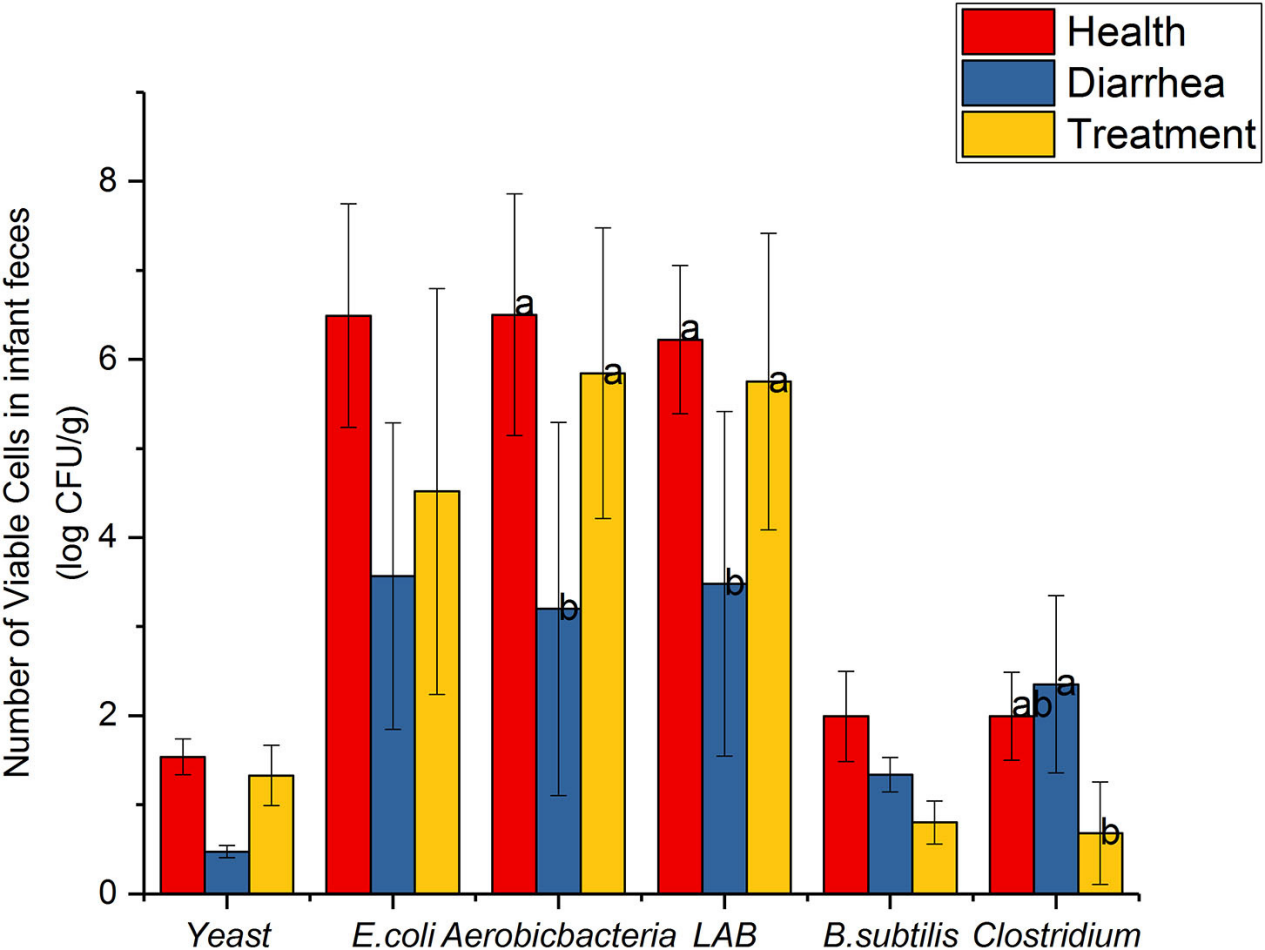
Common viable microbes in infant feces. Different letters above the bars denote statistically significant differences among groups (*P* < 0.05).

Common microbial communities, based on dilution plating are shown (Figure 2). Aerobic-bacteria and LAB strains in diarrhea feces were observably lower than healthy feces (*P* < 0.05), however, cell viability increased after treatment with *L. casei* ZX633, and showed no significant differences with healthy infant feces. *E. coli* and yeast were not remarkably different between diarrhea and healthy feces, but these numbers were lower in diarrhea infant feces. However, these numbers increased and approached the same levels as healthy feces after treatment with *L. casei* ZX633.

### SCFA Levels

**Figure 3:**
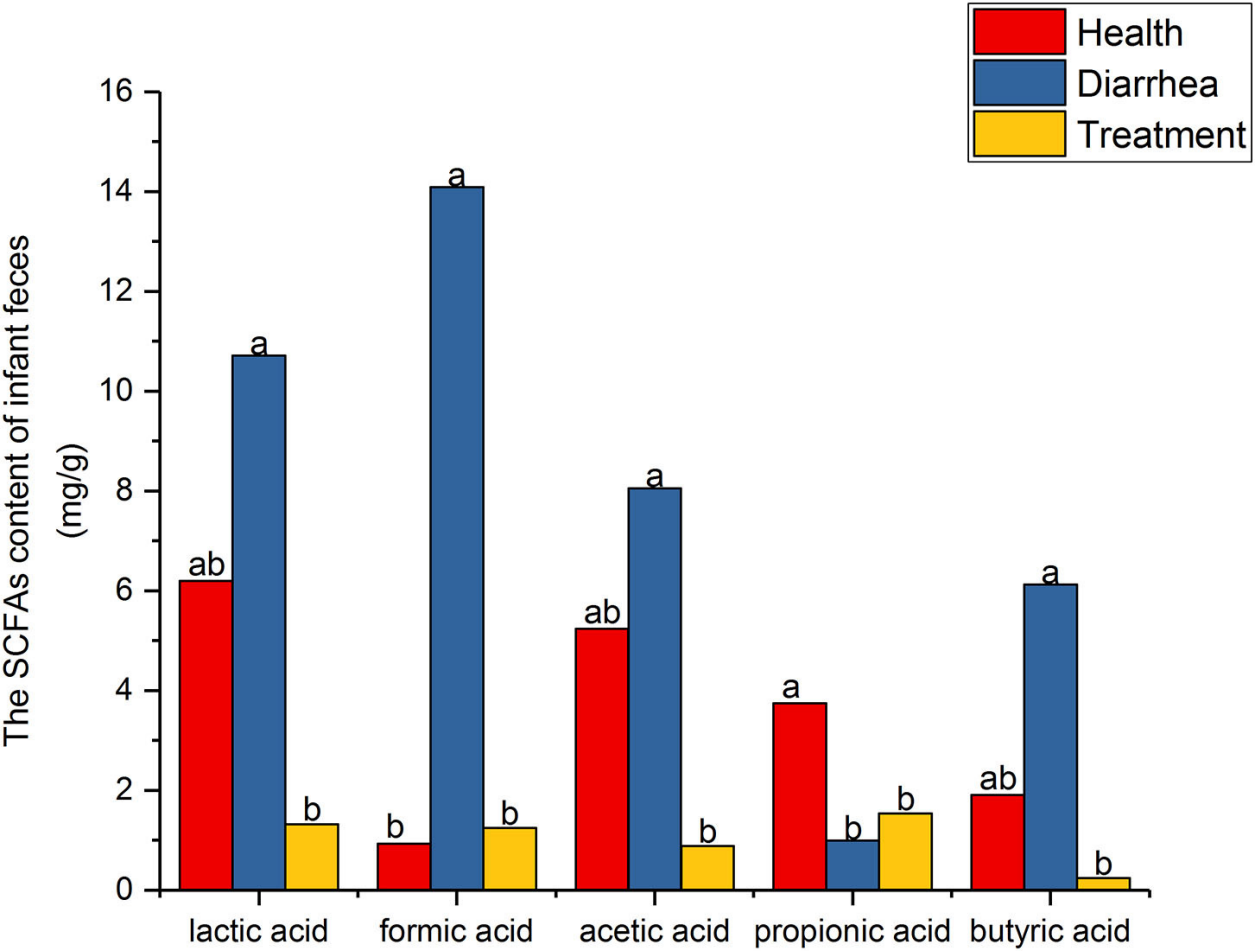
SCFA levels in infant feces. Different letters above the bars denote statistically significant differences among groups (*P* < 0.05).

SCFA level assessment by HPLC is shown (Figure 3). When compared with healthy feces, lactic, formic, acetic and butyric acid levels were higher in diarrhea feces, but were reduced after treatment with *L. casei* ZX633. In contrast, propionic acid levels were significantly (*P* < 0.05) lower in diarrhea feces than healthy feces, but these levels increased after treatment.

### Microbial Diversity in Infant Feces

High throughput sequence analyses of the V3–V4 region of 16S rDNA were used to assess bacterial diversity of infant feces. At shown (Table 2), the mean valid sequences from healthy, diarrhea and treated samples were 48,951, 44,512, and 49,273, respectively. These reads were clustered into 488 OTUs based on 97% sequence identity. The average Good's coverage for samples was >99%, indicating that sequencing depth was adequate for robust sequence analyses.

**Table 2:** Alpha diversity based on OTU levels in infant feces.

**Group**	**Sequence number**	**OTU number**	**Coverage**	**Richness estimator**		**Diversity index**	
				**Chao**	**Ace**	**Shannon**	**Simpson**
Health	48951	136	0.99964	65.80	76.84	1.45	0.36
Diarrhea	44512	312	0.9995	95.18	114.87	1.24	0.47
Treatment	49273	366	0.99958	96.80	111.62	1.17	0.54

Microbial community richness was evaluated based on alpha diversity (Table 2). Compared with healthy group, Chao, ace and Simpson indices were higher, and the Shannon index was lower for diarrhea samples, which signified the abundance for diarrhea samples was higher than healthy samples, but the reverse was diversity. After treating diarrhea samples with *L. casei* ZX633, microbial richness and diversity reduced slightly, as indicated by a lower ace index, and a higher Simpson index.

**Figure 4:**
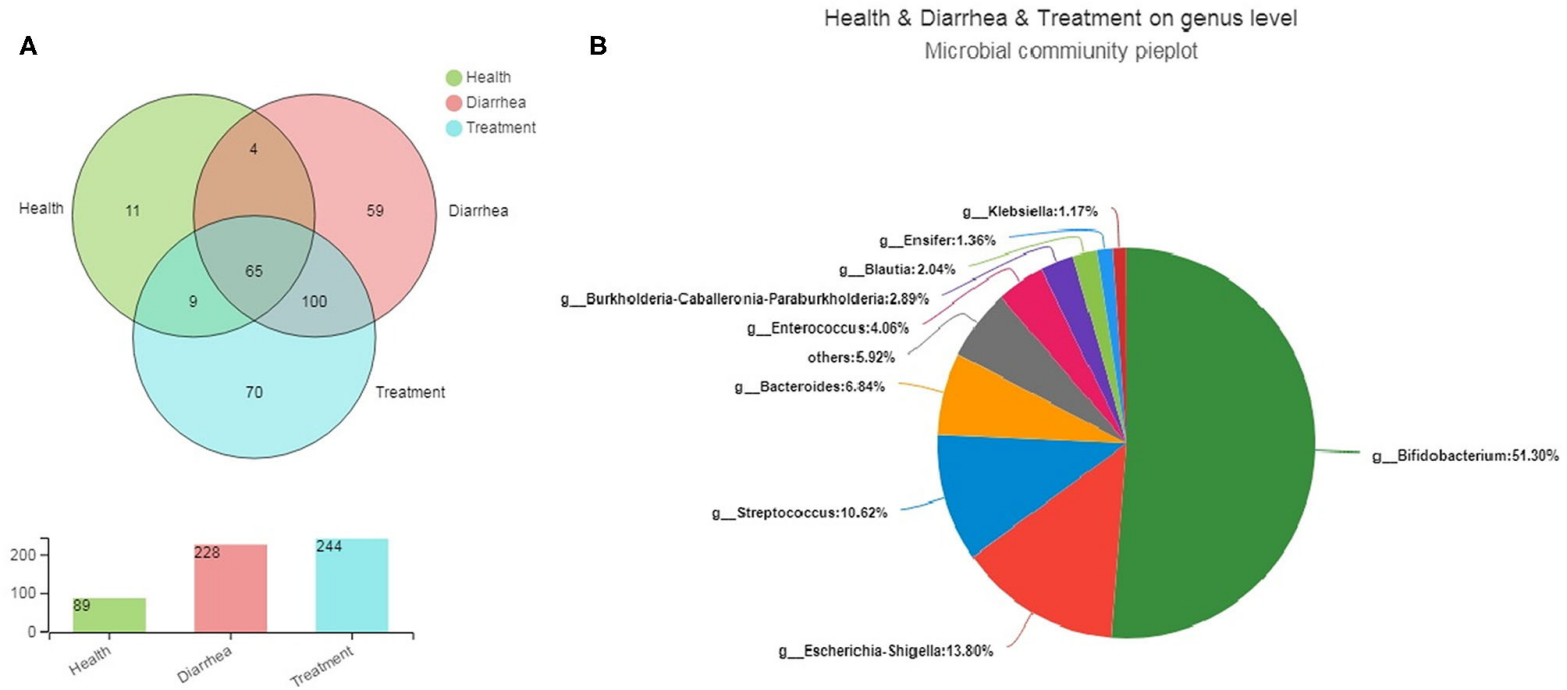
The core microbiota of infant feces at genus levels. **(A)** A Venn diagram showing bacterial numbers shared within and between sample groups. **(B)** Bacterial communities in fecal samples. Less than 1% unclassified genus abundance was merged into “others”.

Bacterial community differences and similarities in fecal groups were analyzed based on genus levels (Figure 4A). We observed 89, 228, and 244 genera in healthy, diarrhea and treated samples, respectively, of which 65 were common genera to all groups, accounting for 20.44% of total observed genera (318). Moreover, 4, 9 and 100 genera were shared by healthy and diarrhea samples, healthy and treatment samples, and diarrhea and treated samples, respectively, whereas 140 unique genera were found in healthy, diarrhea and treatment groups (11, 59, and 70, respectively). Fecal microbiota from the three groups are shown (Figure 4B). The relative genus abundance in all samples was *Bifidobacterium* (51.3%), *Escherichia-Shigella* (13.8%), *Streptococcus* (10.62%), and *Bacteroides* (6.84%). Sequences unclassified into any known groups were specified as “others.”

In fecal samples, sequences were classified into 23 different bacterial phyla, 38 classes, 85 orders, 163 families, 318 genera and 427 species, based on Illumina platform analyses. At the phylum level (Table 3), *Actinobacteria* recorded the highest abundance, and phyla that had relatively high abundance were followed by *Proteobacteria, Firmicutes*, and *Bacteroidetes*. As observed, *Proteobacteria* abundance was largely increased while *Bacteroidetes* were decreased in the treatment group, when compared to the healthy group.

**Table 3:** Relative bacterial abundance at the phylum level in each group.

**Relative abundance (%)**	**Health**	**Diarrhea**	**Treatment**
Actinobacteria	36.51	55.61	55.42
Proteobacteria	33.09	12.66	21.86
Firmicutes	27.34	21.38	14.97
Bacteroidetes	3.01	10.16	7.52
Others	0.05	0.19	0.23

**Figure 5:**
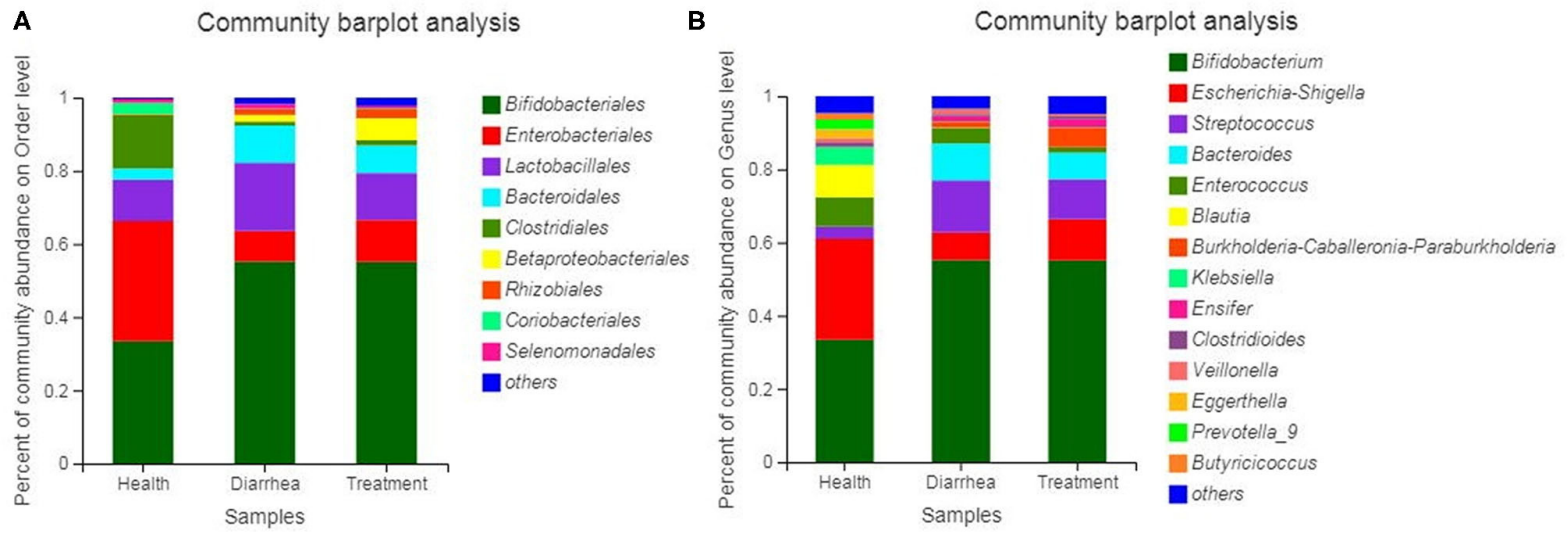
Bacterial community and relative abundance, at the order **(A)** and genus **(B)** levels for all infant feces. Taxa with an abundance <1% are included in “others”.

Bacterial communities based on order and genus level classifications are shown (Figure 5). Microbial composition structures in diarrhea samples differed from healthy samples, which trended toward the healthy group after treatment with *L. casei* ZX633. At the order level (Figure 5A), when compared with healthy group, *Lactobacillales* and *Bacteroidales* abundance was higher and *Enterobacteriales* and *Clostridiales* abundance were lower in the diarrhea group, however these improved after treatment. At the genus level (Figure 5B), *Escherichia-Shigella* and *Clostridioides* abundance increased, and *Streptococcus, Bacteroides, Enterococcus*, and *Veillonella* richness decreased slightly.

**Figure 6:**
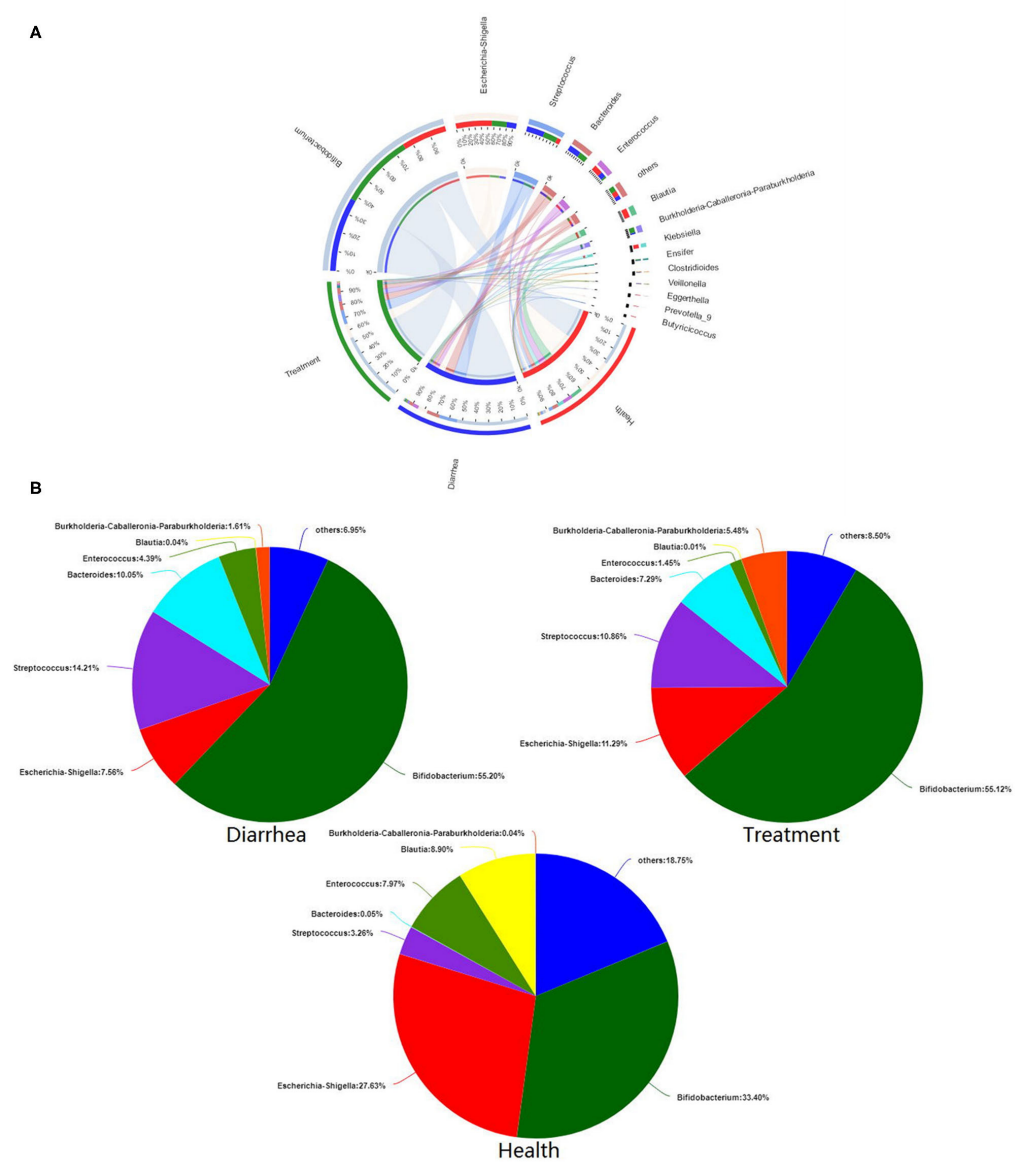
The effects of *L. casei* ZX633 on the gut microbiota of infants with diarrhea. **(A)** A circos plot shows the linkage between samples and bacterial genera. **(B)** Pie charts show gut microbial composition recovery after *L. casei* ZX633 treatment.

Microbial composition and relationships between species and samples showed that predominant bacteria from different groups were similar (Figure 6A). *Bifidobacterium, Escherichia-Shigella, Streptococcus, Bacteroides*, and *Enterococcus* were the five most dominant genera in all fecal samples. The *in vitro* recovery of gut microbial composition after *L. casei* ZX633 treatment is shown (Figure 6B). After treating diarrhea fecal samples with the probiotic, *Escherichia-Shigella* abundance increased from 7.56 to 11.29%, while *Streptococcus* and *Bacteroides* abundance separately decreased, from 14.21 to 10.86% and 10.05 to 7.29%, respectively, which trended toward healthy levels.

### The Relationship Between SCFA Levels and Fecal Bacterial Communities

**Figure 7:**
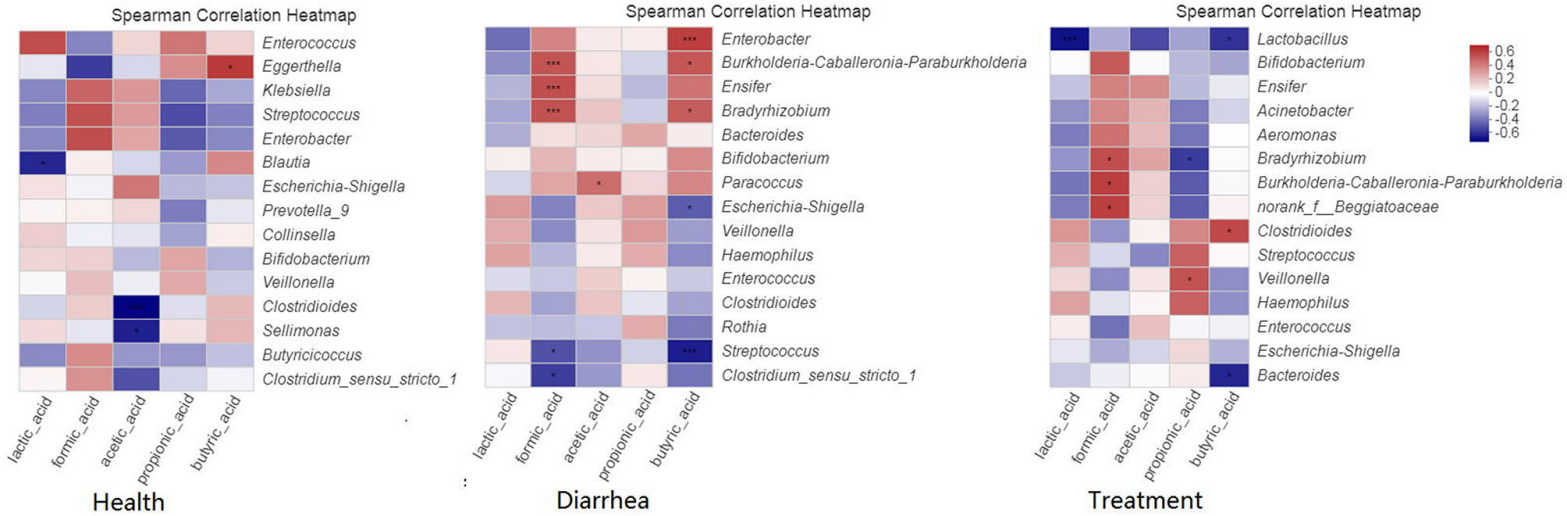
Spearman's correlation heatmaps of the top 15 genera and SCFAs. The X and Y axes are environmental factors and genus, respectively. *P < 0.05, **P < 0.01, ***P < 0.001.

Our correlation heat map showed that the relationship between SCFA levels and microbial composition at the genus level in different infant fecal samples were varied (Figure 7). In the healthy group, butyric acid was significantly (*P* < 0.05) and positively related to *Eggerthella*. In the diarrhea group, formic acid was significantly (*P* < 0.001) and positive correlated with *Bradyrhizobium, Burkholderia-Caballeronia-Paraburkholderia*, and *Ensifer*, and obviously (*P* < 0.05) negatively correlated with *Streptococcus* and *Clostridium*. Acetic acid was positively (*P* < 0.05) correlated with *Paracoccus*, whereas butyric acid was significantly (*P* < 0.001) and positively correlated with *Enterobacter*, negatively (*P* < 0.05) correlated with *Escherichia-Shigella*, and extremely (*P* < 0.001) negatively correlated with *Streptococcus*. In treatment group, formic acid was significantly (*P* < 0.05) and positively correlated with *Bradyrhizobium, Burkholderia-Caballeronia-Paraburkholderia*, and *Beggiatoaceae*, propionic acid showed a notable (*P* < 0.05) negative correlation to *Bradyrhizobium*, and was positively (*P* < 0.05) correlated to *Veillonella*, butyric acid demonstrated a positive (*P* < 0.05) correlation with *Clostridioides*, and a negative (*P* < 0.05) correlation with *Bacteroides* and *Lactobacillus*.

## Discussion

The gut microbiota is a large and complicated ecosystem, with a mutualistic relationship with its human or animal host. The system plays an important role in maintaining gut health and preventing gut infection (Yan et al., 2018). Moreover, there is evidence that microbes at one site can affect the host at other sites, and interesting discoveries on gut microbial community linked with the milieu of the brain, respiratory and urogenital tracts, heart and skin, resulting in that microbial community might be new approaches to health maintenance, disease prevention and even treatment (Reid et al., 2017). Diarrhea is related to an imbalance in the gut microbiota, such as the presence of specific pathogens like *Clostridium* (Mallina et al., 2018), *Salmonella* (Tadesse, 2014), and *Escherichia* (Chingwaru and Vidmar, 2017; Mekonnen et al., 2019). Given the potential resistance effects of some antibiotic therapies for bacterial diarrhea, studies have suggested that probiotics could be an effective strategies in combatting these diarrheal diseases (Szajewska et al., 2011; Hojsak et al., 2018). Hence, we strategically selected *L. casei* ZX633 from healthy feces, and examined its *in vitro* effects on the composition of intestinal microbiota. Since the cultural properties of LAB *in vitro* are crucial to select probiotic strains for treating gut microbiota modulation (Bubnov et al., 2018), we have previously studied the probiotic properties of *L. casei* ZX633 (Wang et al., 2020), including antibiotic sensitivity, aggregation, gastrointestinal survival rate, and so on. Generally, LAB isolated from the human beings are considered to be safe, however, specific research on its internal safety issues will be taken into consideration in further experiments.

LAB are widely distributed in nature. Currently, at least 40 genera are known, mainly comprising *Lactococcus, Lactobacillus, Leuconostoc, Enterococcus, Pediococcus*, and *Streptococcus*. Dairy products contain several genera, including *Lactococcus* (Smit et al., 2005), *S. acidophilus* (Ott et al., 2000), and *Leuconostoc* (Sanchez et al., 2005), whereas *Lactobacillus* (Bernardeau et al., 2008) mainly occurs in plants, animals and silage. *Enterococcus* are widespread in the digestive system of humans and animals (Giraffa, 2002). A previous study showed that *Lactobacillus* was the most prevalent genus in infant feces, followed by *Enterococcus* (Rubio et al., 2014). In this study, *E. faecium* was the most frequently isolated species from infant feces, either healthy or diarrheal, whereas *Lactobacillus* was the second. These observations agree with a previous study showing that *Enterococcus* was the main species isolated from infant feces (Solis et al., 2010).

We also investigated changes in microbiota viability and SCFAs. Some studies have suggested that LAB species contribute to the prevention and treatment of numerous gastrointestinal tract disorders, including diarrhea (Wanke and Szajewska, 2014; Xu et al., 2019). Similarly, *Clostridium* is a commensal gut microbe, and is the foremost cause of nosocomial bacterial infections, making it the most vicious of the enteric pathogens (Kalakuntla et al., 2019). When compared with the diarrhea group, some microbiota species were modulated in the treatment group, such as reductions in *Clostridium* and increased LAB, which could be linked to diarrhea remission. The most abundant microbial metabolites were acetate, propionate and butyrate, each of which acts as a microbial regulatory factor and major modulators of host-microbiome interactions, affecting gut homeostasis and defenses against pathogen invasion (He et al., 2019). *L. casei* ZX633 appeared to exert different effects on SCFA levels, showing reductions in formic acid and increased propionic acid in the treatment group. These findings indicate that *L. casei* ZX633 treatment could improve SCFA levels, and promote gut health in infants with diarrhea, which are closely correlated with the development of microbiota community in diarrhea group toward healthy group.

Soltan et al. reported that LAB were capable of secreting acids, bacteriocins and other by-products that could neutralize infectious pathogens (Soltan Dallal et al., 2017). As a corollary, we observed decreased microbial community richness and diversity after processing feces samples, which may have been attributable to *L. casei* ZX633. In accordance with our data, Lai et al. showed a lower abundance and diversity in the gut microbiota of children, when supplemented with a probiotic *L. casei* (Lai et al., 2019).

According to previous reports, the gut microbiota in infants is enriched with *Bifidobacterium, Escherichia-Shigella, Streptococcus*, and *Bacteroides*, and high levels of *Bifidobacterium* (Yan et al., 2018; Derrien et al., 2019), consistent with our results. Furthermore, we observed a higher abundance of *Escherichia-Shigella* and *Enterococcus*, and a lower abundance of *Bacteroides* in healthy feces, when compared with the diarrhea group. This observation agreed with a recent study showing a decreased abundance of *Escherichia-Shigella* and *Enterococcus*, and an increase abundance in *Bacteroides*, in community-acquired pneumonia in children (Ren et al., 2020). *Bacteroides* can alter gut permeability, increasing immune infiltration, and weakening intestinal epithelial repair (Hua et al., 2016), which eventually promotes the migration of enteric pathogens through the intestinal barrier (Ren et al., 2017). Studies have shown that *Shigella* normally colonizes the human gut and is associated with intestinal infections, such as bacillary dysentery (Anderson et al., 2016), and *Escherichia* causes a broad range of diseases, from diarrheal diseases to extra-intestinal diseases (Walsham et al., 2016; Gallardo et al., 2017). Nevertheless, *Escherichia* is the most common commensal aerobic bacterium in the gut microbiota of humans which had dualism between commensalism and pathogenicity (Massot et al., 2016). Accordingly, an increase in *Escherichia-Shigella* abundance in the treatment group was normal and beneficial. Additionally, *Streptococcus* is frequently isolated from infected abdominal cavities and urogenital systems; the rate of Streptococcal infections is gradually increasing (Wang et al., 2019). Higher *Streptococcus* abundance was found in the diarrhea group, which may possibly indicate infection. After treatment with *L. casei* ZX633, diarrhea was improved as manifested by a reduction in the relative abundance of *Streptococcus*.

The composition and activity of intestinal microbiota influences intestinal environments, and therefore fecal SCFA profiles (Hemalatha et al., 2017), which exert vital influences on intestinal health. SCFAs are important for gut integrity, glucose homeostasis and immune function (Shibata et al., 2017; Liu et al., 2018). Furthermore, the increase in SCFA levels may contribute to the repairs intestinal mucosa, ameliorates permeability, and alleviates diarrhea (Liu et al., 2019). For example, Lee et al. (2017) reported SCFAs mitigate pro-inflammatory responses by inhibiting the NF-κB pathway and may promoted anti-inflammatory cytokine (i.e., IL-10) production. SCFAs are produced by colonic anaerobic microbial communities via the fermentation of indigestible fibrous matter, and some luminal amino acids (Kong et al., 2016). Butyric acid production has been shown to affect the intestines, inhibit pathogen growth, and promote healthy intestinal flora (Liu et al., 2014). In our study, due to changes in SCFA levels and bacterial abundance, correlations between bacterial abundance and SCFAs in different infant fecal conditions were varied.

## Conclusions

In summary, we compared changes in intestinal microbiota in diarrhea samples treated with the probiotic, *L. casei* ZX633. The intestinal flora of diarrhea samples was different to healthy samples, including an increased abundance of some pathogenic bacteria. When supplemented with *L. casei* ZX633, this reduced gut microbiota destruction and promoted the healthy development of intestinal microbial diversity in diarrhea. Our *in vitro* study provides a basis on which to further explore *L. casei* ZX633 as a probiotic for gut microbiota modulation in infants with diarrhea. Further, more comprehensive studies should be performed before these strains are used in clinical practice including the role of inflammation, immunity, intestinal barrier function, and so on.

## Data Availability Statement

The datasets presented in this study can be found in online repositories. The names of the repository/repositories and accession number(s) can be found below: https://www.ncbi.nlm.nih.gov/sra/PRJNA642333, PRJNA642333.

## Author Contributions

XW, MZ, WW, and ZT designed the study and wrote the manuscript. XW, MZ, and YL performed the experiments. XW conducted the statistical and bioinformatics analysis. HL, HZ, and ZT were involved in the revision of the manuscript. All the authors reviewed and approved the final manuscript.

## Conflict of Interest

The authors declare that the research was conducted in the absence of any commercial or financial relationships that could be construed as a potential conflict of interest.
